# Fecal Microbiota Signatures in Celiac Disease Patients With Poly-Autoimmunity

**DOI:** 10.3389/fcimb.2020.00349

**Published:** 2020-07-23

**Authors:** Stefano Bibbò, Marcello Abbondio, Rosangela Sau, Alessandro Tanca, Giovanna Pira, Alessandra Errigo, Roberto Manetti, Giovanni Mario Pes, Maria Pina Dore, Sergio Uzzau

**Affiliations:** ^1^Department of Medical, Surgical and Experimental Sciences, University of Sassari, Sassari, Italy; ^2^Department of Biomedical Sciences, University of Sassari, Sassari, Italy; ^3^Baylor College of Medicine, Houston, TX, United States

**Keywords:** 16S rRNA gene sequencing, celiac disease, gut microbiota, metagenomics, poly-autoimmunity

## Abstract

To date, reliable tests enabling the identification of celiac disease (CD) patients at a greater risk of developing poly-autoimmune diseases are not yet available. We therefore aimed to identify non-invasive microbial biomarkers, useful to implement diagnosis of poly-autoimmunity. Twenty CD patients with poly-autoimmunity (cases) and 30 matched subjects affected exclusively by CD (controls) were selected. All patients followed a varied gluten-free diet for at least 1 year. Fecal microbiota composition was characterized using bacterial 16S ribosomal RNA gene sequencing. Significant differences in gut microbiota composition between CD patients with and without poly-autoimmune disease were found using the edgeR algorithm. Spearman correlations between gut microbiota and clinical, demographic, and anthropometric data were also examined. A significant reduction of *Bacteroides, Ruminococcus*, and *Veillonella* abundances was found in CD patients with poly-autoimmunity compared to the controls. *Bifidobacterium* was specifically reduced in CD patients with Hashimoto's thyroiditis and its abundance correlated negatively with abdominal circumference values in patients affected exclusively by CD. In addition, the duration of CD correlated with the abundance of Firmicutes (negatively) and *Odoribacter* (positively), whereas the abundance of *Desulfovibrionaceae* correlated positively with the duration of poly-autoimmunity. This study provides supportive evidence that specific variations of gut microbial taxa occur in CD patients with poly-autoimmune diseases. These findings open the way to future validation studies on larger cohorts, which might in turn lead to promising diagnostic applications.

## Introduction

Celiac disease (CD) is an immune-mediated enteropathy characterized by multiple pathogenic pathways and triggered by the ingestion of gluten-containing food in genetically predisposed individuals. The disease was once considered a rare condition, while nowadays its prevalence is estimated around 1%, with differences according to the geographical area (Caio et al., 2019). The risk associated with CD depends on genetic variants encoding the HLA-DQ2/DQ8 heterodimers (Sollid et al., 1989). These heterodimers bind and present gluten peptides to CD4+ T cells leading to their activation and triggering a complex immune response, involving both the innate and adaptive immune system. This condition leads to the CD characteristic damage of intestinal mucosa and consequent malabsorption (Di Sabatino and Corazza, 2009). While the HLA loci account for 35% of the risk, at least other 39 non-HLA loci, partly shared with type 1 diabetes (T1D), rheumatoid arthritis, and systemic lupus erythematosus, explain an additional 5% of CD risk, suggesting a shared immunogenetic background of CD with other autoimmune disorders. A number of studies report that CD is frequently associated with poly-autoimmunity (PAI), including diseases such as autoimmune thyroiditis (Hashimoto's thyroiditis, HT), T1D, psoriasis, among others (Guariso et al., 2007; Demirezer Bolat et al., 2015; Canova et al., 2016; Assa et al., 2017). In particular, a study on Sardinian patients has recently shown that about 35% of celiac patients develop at least one further autoimmune disease, confirming a statistically significant association with the propensity to PAI (Bibbò et al., 2017); of note, the Sardinian population provides an ideal setting for the study of autoimmune diseases because of the homogenous and well-preserved genetic background (Sardu et al., 2012).

Genetic predisposition, however, is necessary but not sufficient for CD to develop. As for other immune disorders, epidemiological data suggest that CD risk might be influenced by additional environmental factors, enteric infections, and/or use of antibiotics (Pes et al., 2019). Given the impact of these conditions on the gut microbial communities, there is a growing interest toward the role of the gut microbiota in CD. Furthermore, the gut microbiota has been demonstrated to be essential for the development and maturation of the gut associated lymphoid tissue (Kamada and Nunez, 2014). Therefore, it is not surprising that changes in the taxonomic composition may occur in autoimmune diseases (McLean et al., 2015).

Alterations of the gut microbiota were reported in CD (Cenit et al., 2015; Pes et al., 2019). However, it is still unclear whether the relationships between CD and microbiota changes include disease promotion, causation or other types of association (Sacchetti and Nardelli, 2020). The HLA-DQ2 genotype, by itself, influences the gut microbiota composition being associated with an increased relative abundance of Firmicutes and Proteobacteria and a reduction in *Bifidobacterium* (Olivares et al., 2015). These data suggest that the host genotype, in association with environmental factors, might select for the gut colonizers. On the other hand, the gut microbiome is a highly complex ecosystem with many redundant functions, and it might establish diverse evolutive changes in response to immunogenetic (HLA and non-HLA) and environmental drivers (i.e., food, infections, and drugs), thus influencing the development of CD and other immunological disorders.

The reported association between CD and additional autoimmune diseases prompted the international scientific societies to recommend over time monitoring for the onset of PAI (Ludvigsson et al., 2014). Most of the CD-related PAI diseases (i.e., T1D, HT, psoriasis, and Sjögren's syndrome) show a delayed clinical onset with stealthy pathogenic processes, often characterized by the transition from gut mucosa homeostasis to sub-clinical inflammation, increase of mucosal barrier permeability, and/or altered metabolism (van der Meulen et al., 2016). All these conditions are also expected to affect the ecosystemic balance of the gut microbial communities. Hence, gut microbiota variations in CD individuals with PAI should be investigated to obtain microbial biomarkers enabling PAI pre-clinical diagnosis.

Here, we report the results of a monocentric observational pilot study. Our main purpose was to identify the differences in the gut microbiota composition between CD patients with and without PAI, in order to evaluate the presence of microbial signatures as possible biomarkers of PAI detection in CD patients. Secondly, we aimed at describing the intestinal microbial community changes according to patients and diseases characteristics. Stool was chosen as sample, given the ease of its availability and its relevance as source of potential gut microbial biomarkers.

## Materials and Methods

### Patients Recruitment

Among CD patients regularly followed up in a tertiary teaching center (Clinica Medica, University of Sassari, Italy), 50 subjects were selected for the purpose of the study. CD was diagnosed according to international guidelines (Ludvigsson et al., 2014). Patients were primarily classified according to the presence of additional autoimmune disorders: (i) CD patients with PAI (cases) (PAI-CD), and (ii) CD patients without PAI (controls) (e-CD). Only autoimmune diseases confirmed by specialist certification were considered for the study. Additional inclusion criteria comprised a body mass index (BMI) between 18 and 25 kg/m^2^, duration of the gluten-free diet (GFD) greater than or equal to 1 year, and age ranging from 18 to 60 years.

Exclusion criteria were: (i) intake of antibiotics, probiotics, or prebiotics in the 3 months prior to the enrolment; (ii) a vegetarian or vegan diet; (iii) a history of malignancy, pregnancy, alcohol abuse, or drug addiction.

After clinical evaluation, each patient provided both fecal and blood samples. In addition, three questionnaires regarding anamnestic and anthropometric data, gastrointestinal symptoms, and eating habits and lifestyle were collected. All enrolled patients signed the informed consent to the study.

The study was conducted in accordance with the Declaration of Helsinki, and the protocol was approved by the Local Ethics Committee “Comitato Etico Indipendente, AOU di Cagliari” (Prot. No. PG/2019/4511).

### Sample and Data Collection

A first questionnaire collected anthropometric measurements (height, weight, abdominal circumference), the medical history of CD and autoimmune disorders (reporting the length of time with PAI as the time from the occurrence of the second immune-mediated disorder), medications, and family history. The second questionnaire, the Gastrointestinal Symptoms Rating Scale (GSRS), aimed to evaluate gastrointestinal symptoms in a standardized fashion (Svedlund et al., 1988). In the third questionnaire, based on a protocol previously used to evaluate the type of diet in Italian patients (Buscemi et al., 2015), eating habits were recorded. This food frequency questionnaire investigated the weekly frequency and daily amount of complex carbohydrates, fibers and animal-derived proteins eaten, and the intake of alcohol, coffee and soft drinks; moreover, the fasting time between meals, with attention to night fasting, was recorded. The adherence to GFD was routinely assessed by clinical evaluation, dietetic review, and serum antibodies (transglutaminase IgA/IgG and/or deamidated gliadin peptide IgA/IgG), according to current guidelines (Ludvigsson et al., 2014). The acquisition of data and biological samples and the evaluation of serological markers to confirm GFD took place in the same day. Blood samples were collected in the morning, with the patient fasting for at least 8 hours. The fecal sample was kept at room temperature within a maximum of 6 hours after evacuation. All biological samples were stored at −80°C until processed.

### DNA Extraction and Genomic Characterization

The HLA-DQ2/DQ8 haplotype typing was performed with a standardized method. DNA was extracted from leukocytes, isolated from 6 mL of whole blood in EDTA, using a rapid salting-out protocol with proteinase K. The extracted DNA was dissolved in 1 mM Tris-EDTA, pH 8.0, quantified by UV spectrophotometry at 260/280 nm, and diluted with distilled water at the concentration of 20 ng/μL for PCR. Amplification of the HLA-DQ2/DQ8 alleles was performed by using a specific-allele PCR (ARMS), as previous specified (Sacchetti et al., 1998), with a reported sensitivity and specificity of 98 and 95%, respectively. The following sets of primers DQA1*0501 (5′-AGCAGTTCTACGTGGACCTGGGG-3′ and 5′-GGTAGAGTTGGAGCGTTTAATCAGA-3′), DQB1*0201 (5′-CGCGTGCGTCTTGTGAGCAGAAG-3′ and 5′-GGCGGCAGGCAGCCCCAGCA-3′), and DRB1*04 (5′-GGTTAAACATGAGTGTCATTTCTTAAAC-3′ and 5′-GTTGTGTCTGCAGTAGGTGTC-3′) were used to amplify 144, 110, and 177 bps, respectively.

For the metagenomic analysis, DNA was extracted from fecal samples with the QIAamp Fast DNA Stool Mini Kit (Qiagen, Hilden, Germany). DNA quantification was performed using a Qubit™ Fluorometer with the dsDNA High Sensitivity assay kit (Life Technologies, Carlsbad, CA, USA, now Thermo Fisher Scientific). The variable region 4 (V4) of the gene encoding the 16S rRNA was amplified using the 515F and 806R primers (GTGCCAGCMGCCGCGGTAA and GGACTACHVGGGTWTCTAAT, respectively), modified to contain adaptors for MiSeq sequencing (Illumina, San Diego, CA, USA). The amplified DNA was visualized by electrophoresis performed on 2% agarose gel. Two separate gene amplification reactions were performed for each sample, and the products were pooled together and cleaned up using AMPure XP (Beckman Coulter, Brea, CA, USA) magnetic beads. Libraries were prepared according to the 16S Metagenomic Sequencing Library Preparation protocol, and validated by capillary electrophoresis on a chip, to verify their quality and size, using the BioAnalyzer 2,100 instrument with the Agilent DNA 1,000 Kit (Agilent Technologies, Santa Clara, CA, USA). Subsequently, libraries (average size 445 bps) were quantified with the Qubit dsDNA Broad Range assay kit (Life Technologies) and then normalized. Aliquots of 5 μl of each library were put together to prepare a pool to be loaded in the MiSeq sequencer. Library sequencing was carried out in service using the v3 chemistry, according to the manufacturer's specifications, generating paired-end reads of 201 bases in length in each direction.

### Analysis of Clinical Data

Clinical and anthropometric data, expressed as absolute and percentage numbers, were compared. Statistical analysis was performed using the *t*-test for continuous variables and the Fisher's test for categorical variables by the web application QuickCalcs (graphpad.com/quickcalcs/). The results were considered significant for *p* < 0.05.

### Analysis of 16S rRNA Gene Sequencing Data

Reads quality control and analysis were performed using the Quantitative Insights Into Microbial Ecology (QIIME) pipeline (v.1.9.1) (Caporaso et al., 2010). The overlapping paired-end reads were merged using the script join_paired_ends.py inside the QIIME package. The clustering of the 16S rRNA gene sequences in operational taxonomic units (OTUs) was carried out using the closed-reference OTU picking. OTUs were generated by clustering reads at 97% of identity using UCLUST (Edgar, 2010), and OTUs taxonomic annotation was carried out using the GreenGenes 13_8 database (DeSantis et al., 2006). Alpha-diversity (Shannon and Simpson indexes) and richness (number of observed OTUs) values were calculated after rarefying reads to an equal sequencing depth for all samples, and statistically differences between groups were evaluated with a *t*-test.

Differential analysis was performed on count data at various taxonomic levels, obtained by aggregating OTUs based on their taxonomy assignment, through the MicrobiomeAnalyst web application (http://www.microbiomeanalyst.ca; Dhariwal et al., 2017). Features with prevalence in <10% of samples and coefficient of variation >300% in at least one of the compared groups were filtered out. Count data were transformed prior to statistical testing according to the Relative Log Expression method (Anders and Huber, 2010). Differential abundance analysis was then carried out using the edgeR algorithm (Anders et al., 2013). The *p*-value correction for multiple tests was performed by calculating a False Discovery Rate (FDR) (Benjamini and Hochberg, 1995), and results were considered as significant for FDR <0.05. Finally, Spearman correlation analysis was performed between taxa relative abundance and clinical data, using the *mycor* function on R; the resulting *p*-values were subsequently corrected for multiple tests by calculating an FDR (alpha value = 0.1).

## Results

### Clinical Characteristics of Patients With CD and Other Autoimmune Disorders

A total of 50 CD patients were included in the study between July and October 2018, comprising 20 CD patients with at least another autoimmune disease (PAI-CD, cases) and 30 CD patients without any additional autoimmune disorder (e-CD, controls). The PAI-CD group included patients with one single (*n* =16) or multiple additional autoimmune diseases (*n* = 4). HT was by far the most frequent (*n* = 14) among the additional autoimmune disorders (Table 1).

**Table 1:** Autoimmune disease associated with CD in the PAI-CD group.

	**PAI-CD**
Single additional autoimmune disease	16
Multiple additional autoimmune disease	4
Hashimoto's thyroiditis	14
Type 1 diabetes	3
Sjogren disease	3
Psoriasis	2
Immune thrombocytopenia	2
Myasthenia gravis	1

Gender, age, BMI, abdominal circumference, age at diagnosis of CD, duration of the disease, and familiarity for CD or other autoimmune diseases were similar in both studied groups (Table 2). Moreover, differences for cigarette smoking (20% of smokers in both groups) or use of drugs able to affect intestinal permeability (e.g., anti-inflammatory; Camilleri, 2019) were not detected between cases and controls. Symptoms complained by the patients were also comparable in e-CD and PAI-CD patients according to the GSRS questionnaire (Table 3).

**Table 2:** Characteristics of the fifty patients (20 PAI-CD, 30 e-CD) included in the study.

	**PAI-CD**	**e-CD**	***p*-value**
Gender (M/F)	4/16	2/28	0.20
Mean age (years)	38.6 ± 9.9	39.70 ±10.7	0.71
Body Mass Index (Kg/m^2^)	20.7 ± 2.8	20.3 ± 2.3	0.62
Waist circumference (cm)	69.6 ± 6.7	69.4 ± 8.1	0.83
Age at diagnosis of CD (years)	29.9 ± 11.4	29.2 ± 11.3	0.82
Duration of CD (years)	8.7 ± 6.0	10.5 ± 5.6	0.28
Duration of PAI (years)^*^	9.4 ± 9.3	–	–
Family history of CD (%)	5 (25%)	6 (20%)	0.74
Family history of immune disorders (%)	9 (45%)	10 (33.3%)	0.55

* *These data were recorded in 18 out of 20 patients*.

*For each data comparison the corresponding p-value (t-test for continuous variables and Fisher's test for categorical ones) is reported*.

**Table 3:** Gastroenterological symptoms assessed with the Gastrointestinal Symptoms Rating Scale (GSRS).

	**PAI-CD**	**e-CD**	***p*-value**
Heartburn	0.40 ± 0.50	0.30 ± 0.47	0.47
Upper abdomen discomfort	0.20 ± 0.52	0.33 ± 0.48	0.36
Nausea	0.20 ± 0.41	0.10 ± 0.40	0.40
Bloating	0.60 ± 0.68	0.47 ± 0.57	0.46
Abdominal pain	0.25 ± 0.55	0.33 ± 0.48	0.57
Evacuation frequency	0.95 ± 0.22	0.80 ± 0.41	0.14
Urgency	0.20 ± 0.41	0.23 ± 0.43	0.79

*All symptoms are defined according to a scale based on intensity and frequency, from 0 (symptom absent) to 3 (greater illness). The frequency of evacuations is defined as daily. For each data comparison, the corresponding p-value (t-test) is reported*.

Noteworthy, abuse of soft drinks, coffee, or alcohol was recorded in none of the interviewed CD patients, and all of them followed the GFD strictly, as confirmed by negative serum markers. Differences in the intake of fibers, animal-derived proteins, and complex carbohydrates were not detected between groups. Finally, fasting time spent between dinner or night snack and breakfast was similar in the two groups (Table 4).

**Table 4:** Eating habits.

	**PAI-CD**	**e-CD**	***p*-value**
Coffee (daily)	2.20 ± 1.58	1.97 ± 1.35	0.58
Soft drinks (weekly)	0.55 ± 0.60	0.60 ± 0.56	0.77
Alcoholic beverages (weekly)	0.60 ± 1.57	0.30 ± 0.47	0.33
Milk and dairy (weekly)	5.80 ± 2.07	5.87 ± 1.70	0.90
Meat and fish (weekly)	5.75 ± 1.62	5.33 ± 1.73	0.40
Complex carbohydrates (weekly)	6.90 ± 0.45	6.47 ± 1.28	0.15
Fruits and vegetables (weekly)	6.80 ± 0.52	6.20 ± 1.52	0.09
Night fasting (hours)	10.05 ± 1.36	10.73 ± 1.36	0.09

*The frequency of coffee is defined as daily average, the other foods as a weekly average. Night fasting is defined as the hours spent between the last snack taken before going to bed and breakfast. For each data comparison, the corresponding p-value (t-test) is reported*.

All patients were positive for the HLA-DQ2/DQ8 haplotype. More specifically, the frequency of HLA-DQ2 was 80 and 100% in the PAI-CD cases and in the e-CD controls, respectively.

### Fecal Microbiota of PAI-CD Patients Shows Significant Changes in Dominant Gut Microbiota Taxa

A total of 2,462,056 reads were obtained (49,241 on average per sample) upon amplification and sequencing of the V4 region of the 16S rRNA gene. In order to better inspect the structure and investigate the possible differences occurring between the two groups, the microbiota profile was reconstructed by aggregating the taxonomic data assigned at OTU level. Overall, the most represented phyla were Firmicutes, Bacteroidetes, Verrucomicrobia, Proteobacteria, and Actinobacteria (Supplementary Table S1).

**Figure 1:**
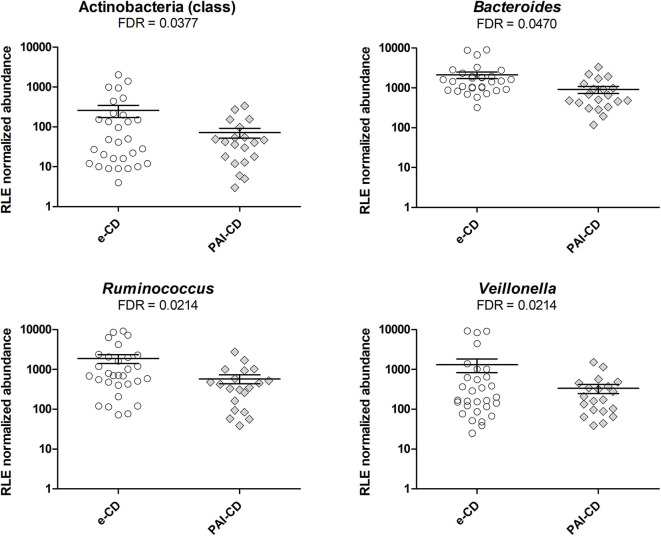
Differential taxa in fecal microbiota of PAI-CD vs. e-CD patients. Scatter plots show taxa with significantly differential abundance between the two groups, after edgeR analysis followed by FDR correction for multiple testing (alpha value = 0.05). Only taxa with abundance >0.5% in at least one group are shown. The FDR value obtained for each taxon is also reported.

**Figure 2:**
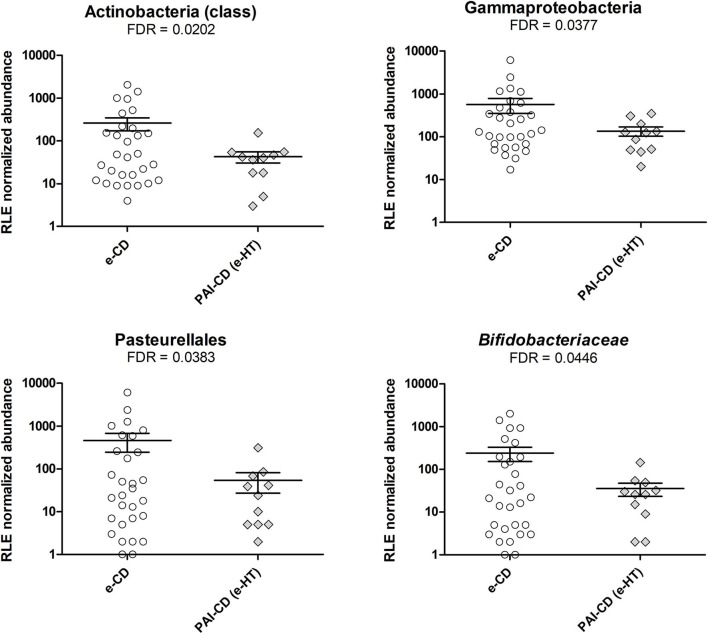
Differential taxa in fecal microbiota of PAI-CD (e-HT) vs. e-CD patients. Scatter plots show taxa with significantly differential abundance between the two groups, after edgeR analysis followed by FDR correction for multiple testing (alpha value = 0.05). Only taxa with abundance >0.5% in at least one group are shown. The FDR-value obtained for each taxon is also reported.

As a first investigation, we compared the gut bacterial composition between e-CD and PAI-CD groups. Upon differential analysis (see Materials and Methods for details), we observed variations at several taxonomic levels (Figure 1). In particular, a significantly lower abundance of the Actinobacteria class was measured in the PAI-CD group. The main genus belonging to Actinobacteria, *Bifidobacterium*, was also decreased in the PAI-CD group, although this difference did not reach statistical significance (data not shown). A low abundant (<0.5%) genus within Actinobacteria, *Adlercreutzia*, also showed a significant reduction in the PAI-CD group, concurring to the overall decrease of its class. Among the most abundant genera, *Bacteroides, Ruminococcus*, and *Veillonella*, were also significantly decreased in the PAI-CD group. When differential analyses were restricted to PAI-CD with HT, we observed again a significantly reduced abundance of Actinobacteria, and besides, of Gammaproteobacteria class and its order Pasteurellales, compared to e-CD individuals. Finally, reduction of *Bifidobacteriaceae* was significant in this subgroup (Figure 2).

Additional analyses performed to detect significant differences according to the genetic background showed a higher abundance of Euryarchaeota and *[Mogibacteriaceae]* in patients (*n* = 4) with an HLA-DQ8 genotype compared to those with an HLA-DQ2 genotype (Supplementary Table S2).

### The Gut Microbiota of e-CD and PAI-CD Patients Shows Changes That Correlate With the Time From Disease Onset and Abdominal Circumference

**Figure 3:**
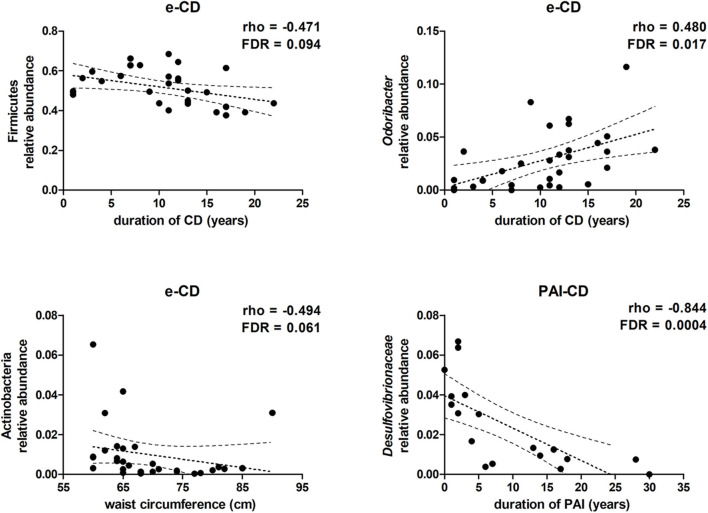
Scatter plots showing correlations between taxa relative abundance and clinical data. Spearman's rho and FDR-values are shown. Dotted lines represent the tendency line, in bold, and the 95% confidence region.

As described above, anthropometric data, gastrointestinal symptoms, eating habits, and lifestyle were similar between e-CD and PAI-CD groups. Within each group, however, we argued that heterogeneity of anthropometric (i.e., BMI and abdominal circumference) and clinical (i.e., time from disease onset) data might correlate with gut microbiota variations. Hence, the gut bacterial composition was further investigated in order to verify this hypothesis. Taking into account the taxa with average relative abundance >0.5%, and established an FDR alpha value of 0.1 for multiple tests, we observed that, in e-CD patients, the duration of CD correlated negatively with the relative abundance of Firmicutes, and positively with that of the genus *Odoribacter* (Figure 3). Furthermore, the length of time with PAI disease was found to correlate significantly with a reduction of *Desulfovibrionaceae* (Figure 3), as well as of the corresponding class and order (Deltaproteobacteria and Desulfovibrionales, respectively).

Moreover, alpha-diversity, according both to Simpson and Shannon indexes, and richness were correlated with the duration of CD. Taking into account all CD patients (*n* = 50), both alpha-diversity and richness increase over time (Supplementary Figure S1). Comparing e-CD vs PAI-CD groups, and e-CD vs PAI-CD patients with thyroiditis as the sole additional disease (e-HT, *n* = 11), alpha-diversity and richness were always higher in e-CD patients, although these differences were not significant (Supplementary Table S3).

In addition, abdominal circumference values were observed to correlate negatively with the relative abundance of the phylum Actinobacteria in e-CD patients (Figure 3), but not in PAI-CD patients (data not shown). On the contrary, no significant correlation was observed with BMI values.

## Discussion

Up to 35% of patients affected by CD, whose prevalence is steadily increasing, are also affected by other autoimmune diseases (Bibbò et al., 2017). Care and clinical management of CD patients, therefore, require biomarkers of PAI risk prediction and/or timing diagnosis criteria to unveil other immunological disorders at their onset. To this purpose, gut microbiota composition and functions might provide useful biomarkers, given that the intestinal inflammatory component is a hallmark of autoimmunity. Indeed, a substantial amount of studies demonstrated the association between autoimmune diseases and gut microbiota composition, yet research on the gut microbiota of individuals with PAI is limited.

Findings from our study confirmed that individuals predisposed to autoimmunity display characteristic features of the gut microbiota. In fact, a significant reduction of *Bacteroides, Ruminococcus*, and *Veillonella* abundances was found in PAI-CD compared to e-CD, as well as *Bifidobacterium* was specifically reduced in HT-CD. In addition, the duration of CD correlated negatively with the abundance of Firmicutes and positively with *Odoribacter*, whereas the abundance of *Desulfovibrionaceae* correlated positively with the duration of poly-autoimmunity.

Several studies reported that gut microbiota variations occur in CD patients, with changes in the relative abundance of certain taxa in comparison to healthy controls (Pes et al., 2019). Such differences appear to be related to the GFD in treated CD individuals, beyond the underlying disease, since the different diets act as inevitable confounding factors. GFD also leads healthy adults to show significant shifts in gut microbiota composition, with a reduction of *Bifidobacterium* and an increase of *Enterobacteriaceae* (De Palma et al., 2009). Noteworthy, genetic background (HLA-DQ2) has been reported to be responsible, per se, for the reduction of *Bifidobacteriaceae* and the increase of *Enterobacteriaceae* in healthy and breast-fed infants (Olivares et al., 2015).

However, our study is the first investigating differences in the gut microbiota in CD patients not biased by important dietary variations such as gluten intake. Hence, to identify PAI-associated changes in the gut microbiota of CD patients, we comparatively investigated patients (with or without PAI), all following a varied GFD from at least 12 months. Besides, the two groups were also quite homogeneous in their genetic HLA-DQ2/DQ8 background. Specifically, HLA-DQ2 had a frequency of 100% among the 30 e-CD patients, while 4 PAI-CD patients presented the HLA-DQ8 haplotype, with the remaining 16 patients being HLA-DQ2+, suggesting a possible role of the DQ8 haplotype in the predisposition to poly-autoimmunity. Indeed, the fecal microbiota of the HLA-DQ8+ subjects showed a significant higher abundance of Euryarchaeota compared to HLA-DQ2+, although the reduced sample size of this pilot study is a limitation to advance any hypotheses.

Our analysis of the fecal microbiota composition revealed some significant differences between the two groups. PAI-CD patients presented a lower abundance of *Bacteroidaceae* family (and of its most relevant genus *Bacteroides*). Members belonging to this microbial family are known to be able to modulate the immune system; in a murine model, *Bacteroides fragilis* was observed to promote the development of Foxp3+ regulatory T cells, favoring the induction of mucosal tolerance (Round and Mazmanian, 2010). In particular, polysaccharide A, which is a symbiotic factor deriving from *B. fragilis*, favors the restoration of a balanced immune response by promoting the secretion of IL-10 (Telesford et al., 2015). Sellitto et al. have also reported a low abundance of Bacteroidetes in infants with a genetic DQ2/DQ8 background (Sellitto et al., 2012). On the contrary, *Bacteroides* was reported to be unchanged in symptom-free CD children on GFD compared to healthy controls, while it appeared increased in symptomatic patients on a normal gluten-containing diet (Collado et al., 2007, 2009; Nadal et al., 2007), although these data were obtained by cultural methods or qPCR target assays. Two other dominant genera within the gut microbiota were found reduced in PAI-CD patients: *Ruminococcus* and *Veillonella*. These two genera comprise well-described propionate-producing species, as also described for *Bacteroides*. This function has been suggested to link the intestinal abundance of these taxa with IL-10-producing regulatory T cell differentiation in gut-associated lymphoid tissues that, in turn, might dampen inflammation (Shimizu et al., 2018).

Our finding about Actinobacteria reduction in PAI-CD patients also deserves attention, since their abundance has been consistently reported as changed in CD patients versus healthy controls (Pes et al., 2019). Both GFD and HLA-DQ2 haplotype have been associated with lower abundance of Actinobacteria and its most prevalent genus *Bifidobacterium* (Cenit et al., 2015). Further, the reduction observed in PAI-CD compared with e-CD patients prompts to speculate that *Bifidobacterium* abundance is inversely associated with the complexity and severity of autoimmune disorders.

Our study was designed to limit the effect of GFD, since both groups of patients examined were on a similar diet for at least 1 year. This also allowed us to examine the correlation of microbiota composition with the duration of CD and the exposure to GFD. In e-CD individuals, the phylum Firmicutes, reported to be increased in individuals with genetic risk for CD (Olivares et al., 2015), showed a reduction of their relative abundance that is directly correlated to the time on a GFD. On the contrary, we observed a positive correlation of time on GFD with increased abundance of *Odoribacter*. The genus *Odoribacter* contains bacterial species known to be butyrate producers. Microbial butyrate is produced by fermentation and represents the most important energy source for intestinal epithelial cells (Roediger, 1980). Butyrate promotes epigenetic-mediated differentiation of regulatory T cells and enhances the maturation and integrity of the intestinal barrier (Peng et al., 2009; Smith et al., 2013). Species within the genus *Odoribacter* have been reported to be decreased in patients with rheumatoid arthritis, in animal models of lupus, and in dextran sulfate sodium-induced colitis (Luo et al., 2018; Sun et al., 2019; Wan et al., 2019). Further, an increase of alpha-diversity and richness, generally referred as directly correlated with a well-balanced microbial ecosystem, were found to be positively correlated with the length of time on GFD, in all patients enrolled in this study. Taken together, the reduction of Firmicutes and the increase of *Odoribacter* abundance and of alpha-diversity, observed following CD diagnosis and GFD introduction, are in line with a progressive return to gut mucosa homeostasis. These results are consistent with a recent study reporting a decrease of *Odoribacter* and alpha-diversity in the gut microbiota of mice (pups) showing poor maturation of the intestinal barrier, increased gut permeability, and inflammation due to a challenged post-natal nutrition (Ley et al., 2019).

When only PAI-CD patients were examined, the abundance of *Desulfovibrionaceae*, a sulfate-reducing bacteria family, was positively correlated with the length of time from the diagnosis of PAI. *Desulfovibrionaceae* is part of the normal gut microbiota, but their increased levels may contribute to colitis development, likely in association with hydrogen sulfide production (Millien et al., 2019). Increase of *Desulfovibrionaceae* has been recently described in patients affected by myasthenia gravis (Moris et al., 2018).

The originality of the study design, which limits the effects of GFD, and the homogeneity of the population, comparable for clinical characteristics and dietary habits, represent the major strengths of this work, which allows us to hypothesize that differences in the gut microbiota could be related to the autoimmune status. On the contrary, the small sample size and the duration of PAI represent its main limitations. Indeed, the lengthy duration of PAI (average duration 9.4 years) did not permit us to investigate about early biomarkers of autoimmunity, but only to hypothesize a signature of undiagnosed subclinical PAI. Instead, our results (in particular regarding the abundance of *Desulfovibrionaceae*) may represent markers of PAI progression, although this hypothesis should be tested in an appropriately designed longitudinal study.

In conclusion, we found evidence, for the first time, of an association between the gut microbiota and PAI in CD patients. According to the experimental evidences of this pilot study, certain variations of the gut microbiota composition might play a role in maintaining the gut homeostasis in GFD-treated CD individuals, while others might promote an increased risk of PAI. More specifically, gut microbiota species belonging to *Bacteroides, Bifidobacterium, Veillonella*, and *Ruminococcus*, as well as to *Desulfovibrionaceae*, warrant further investigation as their modulation might be a potential signature of association with PAI in genetically susceptible hosts. These data might be the basis for new studies designed with a wider number of individuals.

## Data Availability Statement

The datasets presented in this study can be found in online repositories. The names of the repository/repositories and accession number(s) can be found at: https://www.ebi.ac.uk/ena, PRJEB35028.

## Ethics Statement

The studies involving human participants were reviewed and approved by Comitato Etico Indipendente, AOU di Cagliari (Prot. No. PG/2019/4511). The patients/participants provided their written informed consent to participate in this study.

## Author Contributions

SB conceived the study, recruited the patients, collected metadata and samples, and wrote the manuscript. SU conceived the study, supervised the study progress, and wrote the manuscript. MA conducted microbiota analyses, correlation studies and differential analyses, and wrote the manuscript. RS and GP contributed to the microbiota analyses. AE conducted the genetic tests. AT supervised data analysis and reviewed the manuscript. RM, GMP, and MD contributed to the study design. All authors contributed to the article and approved the submitted version.

## Conflict of Interest

The authors declare that the research was conducted in the absence of any commercial or financial relationships that could be construed as a potential conflict of interest.
