# Supplementary material for: Fecal Microbiota Signatures in Celiac Disease Patients With Poly-Autoimmunity
Source: Front Cell Infect Microbiol. 2020 Jul 23;10:349. doi: 10.3389/fcimb.2020.00349 (PMC7390951; doi:10.3389/fcimb.2020.00349)
Supplement: Supplementary file 1 [file Data_Sheet_1.docx]

Supplementary Material

# Supplementary Figures and Tables

## Supplementary Figures

**
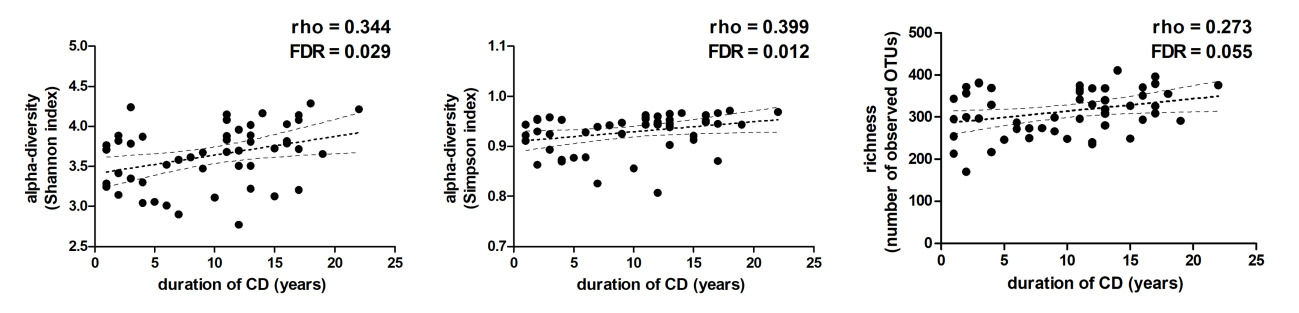
**

**Supplementary Figure S1. Correlation of alpha-diversity and richness with clinical data.** Scatter plots showing correlation of alpha-diversity (both Shannon and Simpson indexes) and richness (number of observed OTUs) values with the duration of CD (years). Spearman's rho and FDR values are shown. Dotted lines represent the tendency line, in bold, and the 95% confidence region.

## Supplementary Tables

**Supplementary Table S1. Phyla distribution among CD patients.** For each patient the relative abundance of the most abundant phyla (top 5) among the study CD cohort was reported. Phylum assignment was performed based on the aggregation of OTUs taxonomic data. For each phylum, mean and standard deviation (SD) are also reported.

| **Sample ID** | **Firmicutes** | **Bacteroidetes** | **Verrucomicrobia** | **Proteobacteria** | **Actinobacteria** |
| --- | --- | --- | --- | --- | --- |
| e-CD_01 | 61.443% | 26.617% | 6.202% | 4.803% | 0.364% |
| e-CD_02 | 39.204% | 51.159% | 0.298% | 0.877% | 6.519% |
| e-CD_03 | 53.712% | 30.017% | 13.511% | 1.214% | 0.118% |
| e-CD_04 | 44.940% | 48.760% | 0.015% | 3.835% | 0.851% |
| e-CD_05 | 43.535% | 22.663% | 26.644% | 2.287% | 4.156% |
| e-CD_06 | 49.168% | 36.147% | 3.827% | 10.386% | 0.042% |
| e-CD_07 | 47.947% | 30.045% | 18.155% | 2.420% | 0.640% |
| e-CD_08 | 43.887% | 46.320% | 0.533% | 6.622% | 1.376% |
| e-CD_09 | 57.087% | 33.426% | 0.109% | 6.781% | 0.806% |
| e-CD_10 | 39.079% | 57.151% | 0.044% | 2.579% | 0.520% |
| e-CD_11 | 49.603% | 44.418% | 0.069% | 3.680% | 0.310% |
| e-CD_12 | 37.682% | 50.336% | 0.119% | 10.744% | 0.178% |
| e-CD_13 | 40.145% | 54.817% | 0.003% | 3.791% | 0.303% |
| e-CD_14 | 43.673% | 49.738% | 2.364% | 2.556% | 0.880% |
| e-CD_15 | 68.445% | 27.072% | 0.857% | 2.453% | 0.666% |
| e-CD_16 | 56.341% | 30.561% | 0.053% | 8.581% | 1.292% |
| e-CD_17 | 57.398% | 38.057% | 0.116% | 1.776% | 0.258% |
| e-CD_18 | 49.218% | 23.686% | 0.008% | 25.427% | 1.199% |
| e-CD_19 | 55.131% | 27.186% | 2.784% | 11.384% | 3.086% |
| e-CD_20 | 66.151% | 24.749% | 0.013% | 8.700% | 0.046% |
| e-CD_21 | 50.093% | 46.436% | 0.016% | 2.757% | 0.442% |
| e-CD_22 | 41.894% | 38.658% | 10.823% | 7.332% | 0.069% |
| e-CD_23 | 56.160% | 34.857% | 0.107% | 5.865% | 0.137% |
| e-CD_24 | 64.437% | 28.796% | 0.153% | 4.927% | 0.281% |
| e-CD_25 | 54.917% | 34.920% | 0.412% | 9.018% | 0.062% |
| e-CD_26 | 49.667% | 47.134% | 0.055% | 2.503% | 0.121% |
| e-CD_27 | 62.818% | 32.029% | 0.027% | 4.489% | 0.207% |
| e-CD_28 | 59.709% | 14.707% | 17.602% | 2.208% | 3.084% |
| e-CD_29 | 43.683% | 17.087% | 37.473% | 1.373% | 0.251% |
| e-CD_30 | 62.753% | 5.241% | 2.410% | 27.466% | 1.408% |
| PAI-CD_01 | 70.385% | 22.147% | 0.024% | 6.707% | 0.198% |
| PAI-CD_02 | 42.452% | 48.254% | 4.699% | 2.927% | 0.963% |
| PAI-CD_03 | 76.060% | 15.417% | 0.269% | 6.933% | 0.169% |
| PAI-CD_04 | 61.958% | 35.004% | 0.013% | 2.210% | 0.357% |
| PAI-CD_05 | 42.217% | 15.518% | 35.242% | 6.150% | 0.112% |
| PAI-CD_06 | 46.521% | 33.504% | 9.711% | 9.514% | 0.404% |
| PAI-CD_07 | 42.220% | 36.503% | 11.481% | 2.428% | 0.807% |
| PAI-CD_08 | 79.656% | 14.208% | 2.893% | 1.867% | 1.004% |
| PAI-CD_09 | 63.051% | 22.534% | 2.642% | 8.561% | 1.240% |
| PAI-CD_10 | 37.065% | 12.722% | 45.072% | 2.014% | 1.642% |
| PAI-CD_11 | 61.900% | 26.425% | 1.542% | 7.962% | 1.819% |
| PAI-CD_12 | 53.200% | 40.368% | 0.035% | 5.375% | 0.518% |
| PAI-CD_13 | 63.858% | 28.120% | 0.448% | 6.738% | 0.741% |
| PAI-CD_14 | 45.007% | 46.386% | 0.028% | 7.958% | 0.289% |
| PAI-CD_15 | 25.507% | 55.625% | 16.143% | 1.853% | 0.229% |
| PAI-CD_16 | 53.171% | 34.009% | 0.010% | 5.563% | 2.113% |
| PAI-CD_17 | 71.738% | 17.940% | 0.248% | 5.501% | 0.332% |
| PAI-CD_18 | 55.873% | 35.132% | 0.042% | 5.725% | 2.533% |
| PAI-CD_19 | 37.284% | 57.805% | 0.088% | 2.885% | 0.239% |
| PAI-CD_20 | 42.351% | 26.015% | 26.903% | 2.481% | 0.182% |
| **mean e-CD** | **51.664%** | **35.093%** | **4.827%** | **6.294%** | **0.989%** |
| **SD e-CD** | **8.841%** | **12.624%** | **9.150%** | **6.270%** | **1.445%** |
| **mean PAI-CD** | **53.574%** | **31.182%** | **7.877%** | **5.068%** | **0.795%** |
| **SD PAI-CD** | **14.682%** | **13.569%** | **13.146%** | **2.522%** | **0.722%** |
| **mean total** | **52.428%** | **33.528%** | **6.047%** | **5.804%** | **0.911%** |
| **SD total** | **11.434%** | **13.018%** | **10.901%** | **5.109%** | **1.203%** |

**Supplementary Table S2. Differential taxa in fecal microbiota of HLA-DQ8+ vs HLA-DQ2+ patients.** Taxa with significantly differential abundance between the two groups, after edgeR analysis followed by FDR correction for multiple testing (alpha value = 0.05), are reported, together with the logarithm of the fold change (logFC). Taxa with abundance >0.5% in at least one group are shown.

| **taxon** | **logFC**  **DQ8+/DQ2+** | **FDR (0.05)** |
| --- | --- | --- |
| Euryarchaeota | 3.57 | 0.000013 |
| *[Mogibacteriaceae]* | 2.40 | 0.042186 |

**Supplementary Table S3. Alpha-diversity and richness within fecal microbiota of e-CD, PAI-CD, and PAI-CD (e-HT) patients.** Alpha-diversity (Simpson and Shannon indexes) and richness (number of observed OTUs) values and their respective standard deviations are showed. No statistically significant differences between groups, according to *t*-test, were found.

| **Sample group** | **alpha-diversity** | | **richness** |
| --- | --- | --- | --- |
|  | **Simpson** | **Shannon** |  |
| e-CD | 0.938 ± 0.032 | 3.690 ± 0.345 | 314.4 ± 51.9 |
| PAI-CD | 0.915 ± 0.045 | 3.549 ± 0.435 | 311.2 ± 61.2 |
| PAI-CD (e-HT) | 0.912 ± 0.040 | 3.435 ± 0.381 | 290.7 ± 66.2 |
